# Persistent postoperative pediatric pain: a secondary analysis of the 4P prospective observational multicenter study

**DOI:** 10.3389/fpain.2026.1930534

**Published:** 2026-10-05

**Authors:** Anna K. M. Persson, Johanna Broman

**Affiliations:** 1 Department of Anesthesiology and Intensive Care Medicine, Halland Hospital Halmstad, Halmstad, Sweden; 2 Department of Clinical Sciences Malmö, Lund University, Malmö, Sweden; 3 Department of Anesthesiology and Intensive Care, Helsingborg Lasarett, Helsingborg, Sweden; 4 Department of Clinical Sciences Helsingborg, Lund University, Helsingborg, Sweden

**Keywords:** chronic pain, pain after surgery, pediatric pain, persistent pain, postoperative pain

## Abstract

**Introduction:**

Few large studies have examined the prevalence and risk factors for persistent postoperative pain (PPOP) in children. Chronic pain in childhood can lead to significant personal suffering, including non-attendance at school and social isolation.

**Methods:**

In a prospective observational multicenter study, we followed 601 children undergoing common pediatric surgeries. This secondary analysis aimed to describe the prevalence, characteristics, and exploratory risk factor associations of PPOP. The primary outcome was defined as recurrent pain (4 on an age-specific scale lasting for over three months after surgery. Persistent pain was further characterized regarding its impact on various activities and whether it was anatomically localized at the site of surgery.

**Results:**

Of the 601 children, 380 were available for follow-up at 3 and 6 months after surgery. The median age was 5 years, and 34% were female. Preoperative pain was reported by 41 children (11%), and 126 (33%) reported anxiety or sleep problems before surgery. Thirteen (3%) patients experienced PPOP (≥ 4) after either 3 (*n* = 9) or 6 months (*n* = 4). Ten attributed the pain to the surgical area, and in six children, the pain affected activity-levels. Five children used analgesics for their symptoms. Only one child experienced pain daily, and three children reported weekly pain. Applying a stricter definition from ICD-11 to our cohort resulted in an even lower PPOP prevalence: only three (0.8%) children experienced pain lasting more than 3 months, in whom the pain began or increased after surgery, was at a level ≥4, influenced activity-levels, and was located in the surgical area.

**Conclusion:**

The prevalence of persistent postoperative pain after common pediatric surgeries was very low. As one of the few prospective multicenter studies in this field, these findings are noteworthy.

## Introduction

In adults, the prevalence of persistent postoperative pain (PPOP) ranges from 5% to 50% (1). In children, far fewer studies exist, and reported prevalence varies even more (2, 3). A 2017 meta-analysis identified only four studies and estimated an overall prevalence of about 20% across mixed surgical procedures, emphasizing the urgent need for more research (2). Only two of the included studies assessed pain at 3 and 6 months after orthopedic and general surgery, reporting prevalences of 10.9% and 23%, respectively (4, 5); the other studies reported pain after spinal fusion.

In 2018, Mosetti et al. prospectively evaluated PPOP after common pediatric surgery (*n* = 350), defining it as intermittent or constant pain ≥4 lasting more than 3 months. Pain was assessed using age-appropriate 0–10 pain scales (FLACC scale < 3 years, Wong-Baker Faces 3–6 years, NRS >6 years). They also evaluated effects on daily activities, sleep, and other complications. Prevalence was 6% at 3 months and 4% at 6 months. No association was found between moderate- to severe acute postoperative pain (APOP) in the postanesthesia care unit (PACU) and persistent pain (6). Other studies have reported higher prevalences. In 2011, in a cross-sectional study, Fortier et al. conducted structured telephone interviews with children aged 2–17 years who had undergone general, urologic, or orthopedic surgery within the previous 10 months. Fifteen of 113 children (13%) reported current surgery-related pain, with a mean intensity of 4.2 on NRS, and interference with daily activities (7). One of the few recent prospective studies is a 2025 Spanish investigation of risk factors for PPOP after common pediatric surgery in children aged 4–7 years. Using a definition of pain ≥4 at 3 or 6 months, they reported markedly higher prevalences: 35% at 3 months and 27% at 6 months (8). None of these previous studies has used the ICD-11 definition of PPOP: pain persisting for more than 3 months after surgery, pain that begins or worsens after surgery, is located in the surgical area, is associated with pain-related distress, and interferes with daily activities (9).

A recent meta-analysis published during the completion of this study estimated the prevalence of PPOP in the pediatric population at a remarkably high 28% following major surgery. The definition of PPOP used in the included studies was pain at different levels lasting more than 3 months after surgery. None of the included studies used the ICD-11 definition of PPOP. The high prevalence of PPOP found highlights the inconsistency of current evidence, emphasizing the need for large prospective studies (10).

A considerable proportion of children (approximately 15% to 25%) experience persistent pain in daily life, which can lead to significant personal suffering, including school non-attendance and social isolation, and represents a serious public health concern (3, 11). Among hospitalized children, 24%–27% report moderate to severe pain, most surgery-related (12, 13), and as many as 80% self-report current pain during hospitalization (14). Pain management in children is frequently inadequate (15–17), and those who develop long-term pain appear to be at greater risk of ongoing pain-related problems than adults (2). Since surgery is a potential trigger for pain, PPOP is a critical issue to address in pediatric care (3).

Regarding risk factors for persistent pain, gender differences have been observed in childhood pain conditions such as headache and abdominal pain, with girls reporting more pain than boys (11). Clinical studies on postoperative pain are consistent with this pattern, suggesting that both APOP and PPOP are more common in girls (18). Regarding age, some studies suggest that younger children have a lower risk of PPOP (3, 8, 15), whereas other studies find no protective effect once confounders are accounted for (5). Psychological factors also appear influential. Children are highly affected by caregiver behavior, and elevated parental anxiety surrounding surgery has been identified as a potential risk factor (19). Parental catastrophizing, mental health problems, and chronic pain have similarly been associated with increased pain in children (20, 21). Evidence suggests that parental influence on a child's pain response may even increase as more time passes after surgery (21).

The 4P study (Persistent Postoperative Pediatric Pain) is a prospective observational multicenter study following children undergoing common pediatric surgeries in Sweden between 2023 and 2024. This predefined secondary analysis focuses specifically on PPOP with the aim of describing the prevalence, characteristics, and exploratory risk factor associations of PPOP. The primary outcome was defined as recurrent pain ≥4 lasting for over 3 months after surgery. Persistent pain was further characterized regarding its impact on various activities and whether anatomically localized at the site of surgery, and we also present prevalences based on the ICD-11 definition of PPOP. By examining in detail the small subgroup of children who develop PPOP and describing the characteristics that distinguish them, this analysis aims to improve our understanding of why some children develop PPOP while others do not.

## Materials and methods

### Patients

This study was approved by the Swedish Ethics Review Authority on December 1, 2022 (Dnr 2022-04821). The research protocol was preregistered at clinicaltrials.gov under NCT06035042, and a study protocol has been published (22). Children aged 1–17 years scheduled for one of the ESPA-defined pediatric surgical procedures (inguinal hernia, circumcision/hypospadias/cryptorchidism, adenoidectomy/tonsillectomy/tonsillotomy, appendectomy, acute orthopedic fracture surgery) were recruited from 10 hospitals in southern Sweden in 2023–2024. As this was an observational study, the methods used for anesthesia and analgesia, as well as surgical techniques, varied according to local practice. Exclusion criteria included: repeated surgery, inability of the child or caregiver to speak/read Swedish, and inability to understand the implications of participation. Patients aged 15 years or older provided written and oral consent themselves. For younger children, caregivers provided consent after receiving written information a few days before surgery, and in-person information upon hospital arrival. For participants under 15 years, consent from both legal guardians was required, in accordance with Swedish Ethical Review Authority regulations.

### Data collection

PPOP was documented using the data management software Entermedic (Entergate ®, Halmstad, Sweden), which was programmed to send automated text-message questionnaires at the prespecified follow-up time points of 3 and 6 months after surgery, including reminders when necessary. All participants received two reminders to answer the follow-up. Thereafter, responding was not possible, as the time frame for follow-up would have been misleading, and the responses were then handled as missing values. The questionnaires answered were complete. Entermedic is certified according to the international security standard (ISO 27000:1) and is completely adapted to the European Union's requirements of the General Data Protection Regulation. The service is also adapted to comply with Swedish law for patient data reporting, holding, and management (Department of Social Affairs number 2008:335, 2010:659, and 1985:562). Furthermore, Entermedic is approved by the Swedish Medical Products Agency.

### Outcome measurement

Before surgery, all legal guardians (or patients aged 15 years and older) completed a questionnaire addressing preoperative pain: recurrent pain before surgery (yes/no), pain intensity (FLACC, FPS-Revised, or NRS depending on age), duration of pain, and whether it affected normal activities. We also asked whether the child had experienced pain in the 24 h prior to surgery (yes/no) and whether analgesics had been offered. Sleep quality and anxiety were assessed similarly with a yes/no question, including duration and impact on daily activities. Self-reported parental stress (NRS 0–10) was recorded at 24 h, 3 and 6 months after surgery. The levels of stress at the time of the respective pain measurements were used for this analysis. Levels ≥4 were used when dichotomizing the stress level for logistic regression.

At 3 and 6 months postoperatively, a text-message questionnaire was sent to assess current pain using the same age-appropriate scale introduced at the hospital visit. For children aged 1–3 years, we used the FLACC (Face/Legs/Activity/Cry/Consolability Pain Scale, 0–10) (23); for ages 4–14, the FPS-Revised (The Faces Pain Scale-Revised, 0–10) (24); and for ages 15–17 the NRS (Numeric Rating Scale, 0–10) (25). Higher scores indicated more pain, with zero representing “no pain.” The follow-up questionnaire also included questions about pain characteristics, its impact on activity level, and the need for analgesics. For patients under 15 years of age, the legal guardians answered the questionnaires and performed the pain measurements together with their child.

### Definition of PPOP

PPOP was evaluated at 3 months and 6 months after surgery. The definition of PPOP varies across studies (12, 26). In the 4P study, PPOP was primarily defined as any worst pain score ≥1 (FLACC, FPS or NRS), while moderate to severe PPOP was defined as pain ≥4 accompanied by reported impact on activity level and the need for analgesics (22). For this predefined secondary analysis, we applied the cutoff of ≥4 to define PPOP, aligning with thresholds used in other recent pediatric studies (4–6, 8). Using the same definition facilitates comparison across studies and increases the value of future meta-analyses.

We also applied a modified ICD-11 definition (not yet implemented in Sweden): pain persisting for more than 3 months after surgery, pain that begins or worsens after surgery, is located in the surgical area, and interferes with daily activities in this cohort (9). We did not include pain-related distress as this was not measured.

### Statistics

The primary outcome in the 4P study was descriptive and hypothesis-generating, therefore, no formal power calculation was performed. Previous studies (5) suggest that approximately 20% of patients may experience PPOP or APOP. Based on this, we aimed to include 1,000 children to allow analysis of five risk factors (preoperative pain, anxiety/sleep disorder, age, gender, and parental stress).

As pain scales are nonlinear and ordinal, and the outcome event rate was low, we used nonparametric tests. Data are presented at the group level using descriptive statistics, reported as medians with ranges or interquartile ranges (IQR) when appropriate. Dichotomous variables are presented as percentages. Group comparisons were performed using the Mann–Whitney U test for continuous variables and the chi-square test for dichotomous variables. Proportions were compared using the chi-square test when cell counts were over 5 in both groups and Fisher's exact test when cell counts were ≤5. A univariable logistic regression model was constructed to evaluate risk factors for PPOP, using PPOP ≥4.0 as the dependent variable. Because only 13 outcome events occurred, the number of events was insufficient for reliable stepwise multivariable logistic regression. Therefore, analyses were restricted to univariable models.

Parental stress was defined as present if graded ≥4 at either 3 or 6 months. APOP was defined as present if graded ≥4 at the respective time point. Group comparisons are reported as *P* values, with the margin for a type 1 error set to 0.05, in conjunction with the 95% CI. For statistical analyses, IBM SPSS Statistics Version 28.0.0.0 was used.

## Results

### Participants

Out of 1,461 eligible children, 601 were included in the study, and 380 were available for follow-up regarding PPOP at either 3 or 6 months. At the 3-month follow-up, 346 participants responded. Of these, 71 did not respond at 6 months, while 34 participants who had not replied at 3 months completed the 6-month questionnaire, resulting in 309 responses at the 6-month follow-up. Thus, 275 children responded at both time points and 105 at only one time point (Figure 1).

**Figure 1:**
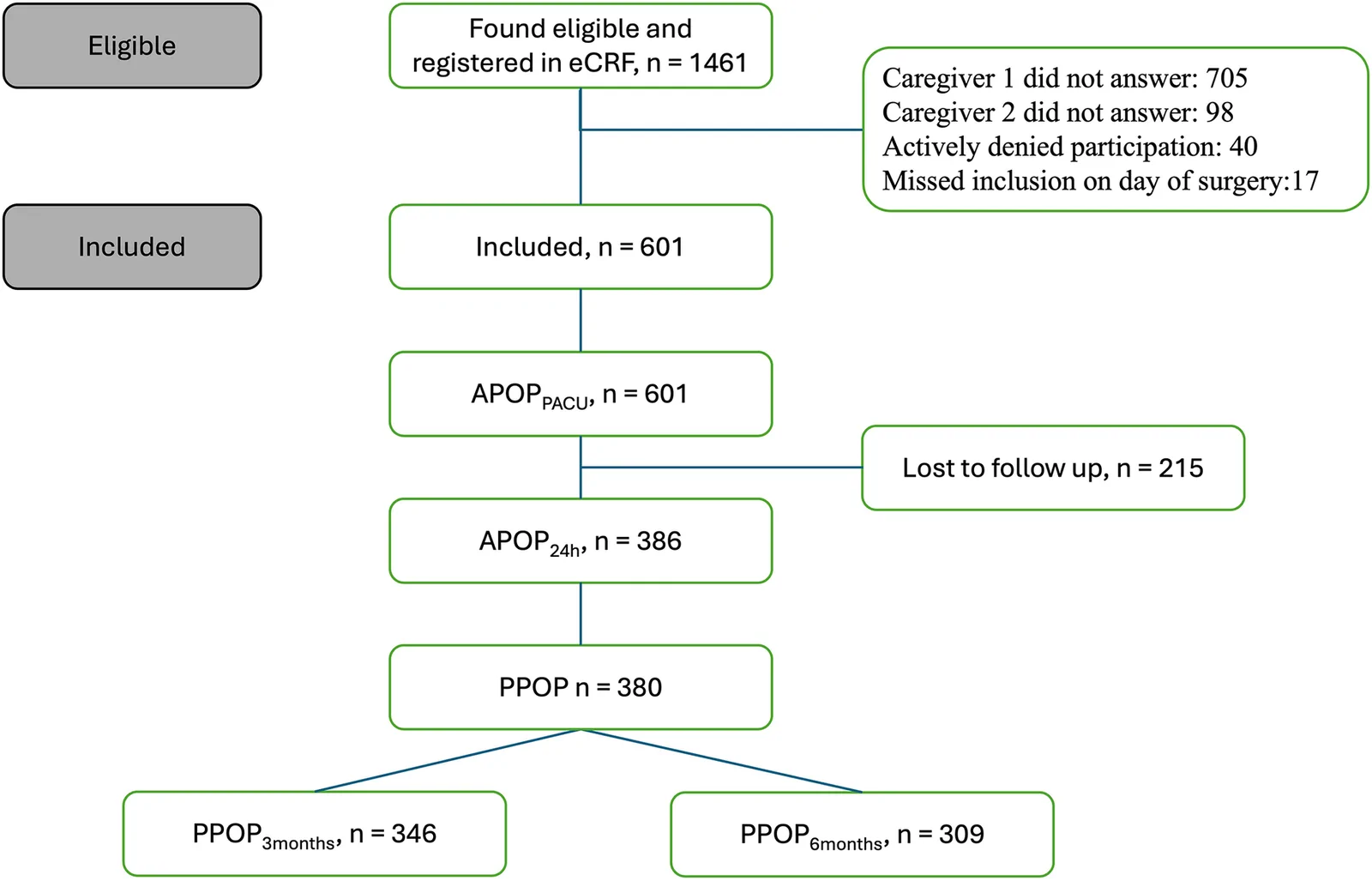
Inclusion flow chart. eCRF, electronic case report form; APOP, acute postoperative pain; PPOP, persistent postoperative pain.

There were no differences in age (4 vs. 5 years, *p* = 0.309), gender (34% vs. 34% females, *p* = 0.894) or anxiety/sleep disturbance (32% vs. 33%, *p* = 0.708) between responders and non-responders. Non-responders had slightly higher prevalence of preoperative pain (18% vs. 11%, *p* = 0.017).

### Patient characteristics

The median age of the cohort was 4 years, and 34% were female. Preoperative pain was reported by 41 children (11%), 28 had experienced pain for more than 3 months, 12 for more than 2 weeks but less than 3 months, and in 25 cases, the pain was significant enough to affect activity-levels. A total of 126 children (33%) reported preoperative anxiety or sleep problems; 65 reported these problems for more than 3 months, and 61 reported them only in the days immediately before surgery. In 37 children, the anxiety was severe enough to interfere with daily activities. Most participants (57%) underwent elective adenoidectomy, tonsillectomy, or tonsillotomy. Additional background characteristics are presented in Table 1.

**Table 1:** Background information and PPOP prevalence, comparing the full 4P cohort with the children included in this study 3–6 months after surgery.

Variable	All patients, *n* = 601	All responders to PPOP follow-up, *n* = 380	Responders after 3 months, *n* = 346	Responders after 6 months, *n* = 309
Age at inclusion, median years (range)	5.0 (1–16)	4.0 (1–16)	4.0 (1–16)	4.0 (1–16)
Female, *n* (%)	206 (34%)	131 (34%)	118 (34%)	106 (34%)
Preoperative pain, *n* (%)	80 (13%)	41 (11%)	37 (11%)	34 (11%)
Anxiety or sleep disturbance, *n* (%)	196 (33%)	126 (33%)	113 (33%)	96 (31%)
Type of surgery, *n* (%)				
ENT	341 (57%)	194 (51%)	177 (51%)	155 (50%)
Appendectomy	31 (5%)	25 (7%)	24 (7%)	20 (6%)
Inguinal hernia	39 (6%)	26 (7%)	26 (8%)	22 (7%)
Circumcision	19 (3%)	11 (3%)	10 (3%)	9 (3%)
Retentio testis	35 (6%)	25 (7%)	23 (7%)	24 (8%)
Fracture surgery	110 (18%)	84 (22%)	72 (21%)	65 (21%)
Hypospadia	26 (4%)	15 (4%)	14 (4%)	14 (5%)
PPOP, *n* % (95% CI)		13/380, 3% (2.0%–5.8%)	9/346, 3% (1.4%–4.9%)	4/309, 1% (0.5-%-3.3%)

ENT, ear, nose, and throat.

### Prevalence and characteristics of PPOP

Among the 13 children with PPOP ≥4, 10 localized the pain to the surgical area and 3 to another area. In six cases, the pain was severe enough to limit daily activity-levels. Five children required analgesics. Only one child reported daily pain, and three reported weekly pain. None had PPOP ≥ 4 at both the 3- and 6-month follow-ups, even though half of those with PPOP responded at both time points.

Preoperative characteristics of children with and without PPOP are presented in Table 2.

**Table 2:** Preoperative data and data on acute postoperative pain for patients with and without PPOP ≥4.0 at either 3 or 6 months after surgery and without.

Variable	Total	No PPOP	PPOP ≥ 4	*P*-value
Number of patients	380	367	13	
Surgery type, *n* (%)				
ENT	194 (51%)	190 (52%)	4 (31%)	
Appendectomy	25 (7%)	25 (7%)	0 (0%)	
Inguinal hernia	26 (7%)	22 (6%)	4 (31%)	
Circumcision	11 (3%)	10 (3%)	1 (8%)	
Retentio testis	25 (7%)	25 (7%)	0 (0%)	
Fracture	84 (22%)	81 (22%)	3 (23%)	
Hypospadia	15 (4%)	14 (4%)	1 (8%)	
Female gender, *n* (%)	131 (34%)	128 (35%)	3 (23%)	0.555
Age, median years (range)	4.0 (1–16)	4.0 (1–16)	2.0 (1–16)	0.498
Preoperative pain, yes/no, *n* (%)	41 (11%)	37 (10%)	4 (31%)	0.041
> 3 months, *n* (%)	28	25	3	
> 2 weeks, *n* (%)	12	11	1	
Influence on activities, *n* (%)	25	21	4	
Preoperative sleep disturbances or anxiety, *n* (%)	126 (33%)	123 (34%)	3 (23%)	0.557
Regional block, *n* (%)	41 (11%)	36 (10%)	5 (38%)	0.001
Feeling of pain being well treated during surgery, *n* (%)	310 (82%)	302 (82%)	8 (62%)	0.382
Experienced breakthrough pain in the PACU, *n* (%)	95 (25%)	92 (25%)	3 (23%)	0.723
APOP in the PACU, median (IQR)	0.0 (0.0–3.0)	0.0 (0.0–3.0)	0.0 (0.0–1.5)	0.155
APOP at 24 h, median (IQR)	1.0 (0.0–2.0)	1.0 (0.0–2.0)	3.0 (2.0–3.0)	<0.001
Rescue opioids in the PACU, *n* (%)	61 (16%)	58 (16%)	3 (23%)	0.447
Self-reported parental stress at 3 months, median (IQR)	0.0 (0.0–2.0)	0.0 (0.0–2.0)	6.0 (5.0–7.5)	<0.001
Self-reported parental stress at 6 months, median (IQR)	0.0 (0.0–1.0)	0.0 (0.0–1.0)	4.0 (2.5–7.5)	<0.001
Self-reported parental stress ≥ 4, *n* (%)	72 (19%)	62 (17%)	10 (77%)	<0.001

ENT, ear nose and throat; PPOP, persistent postoperative pain; APOP, acute postoperative pain; PACU, postanesthesia care unit; SD, standard deviation; IQR, interquartile range.

### Risk factors for persistent postoperative pain

In children developing PPOP, a higher proportion reported preoperative pain compared with those who did not develop PPOP (31% vs. 10%, *p* = 0.018) (Table 2). There were no differences in gender or age between the groups. A higher proportion of children in the PPOP group had received a regional block (38% vs. 10%, *p* = 0.001), although not all procedures routinely allow regional anesthesia. Median APOP at 24 h was also higher among children who later developed PPOP (3.0 vs. 1.0, *p* < 0.001). The proportion of children with high parental stress levels was higher in the group developing PPOP (77% vs. 17%, *p* < 0.001) (Table 2).

In the univariate logistic regression, the only statistically significant risk factors associated with PPOP ≥4 were preoperative pain (OR 4.0, 95% CI 1.2–13.5, *p* = 0.028) and parental stress (OR 16.4, 95% CI 4.4–61.3, *p* < 0.001) (Table 3).

**Table 3:** Univariate logistic regression with PPOP ≥ 4 as the dependent variable.

Variable	OR	95% CI	*P*-value
Gender	0.6	0.2–2.1	0.385
Preoperative pain	4.0	1.2–13.5	0.028
Age	1.0	0.9–1.2	0.698
Preoperative anxiety or sleep problems	0.6	0.2–2.2	0.437
Parental stress ≥ 4	16.4	4.4–61.3	<0.001
APOP in the PACU ≥ 4.0	0.6	0.1–2.9	0.542
APOP at 24 h ≥ 4.0	1.4	0.2–11.6	0.756
Rescue opioids in the PACU	1.6	0.4–6.0	0.486

APOP, acute postoperative pain; PPOP, persistent postoperative pain; PACU, postanesthesia care unit, CI; confidence interval, OR; odds ratio.

### General description of the patients with PPOP

None of the affected children had PPOP ≥ 4 at both the 3- and 6-month follow-ups, even though half responded at both time points. Four of the 13 children underwent open inguinal hernia repair. Two of them received regional blocks. Four children reported preoperative pain, all of them to the extent that they experienced pain severe enough to limit daily activity. Three patients experienced breakthrough pain in the PACU. One legal guardian felt that their child did not receive adequate analgesia during and after surgery. Ten children had a pain score of 0 recorded in the PACU, and three required rescue opioids. Only one child reported APOP ≥ 4 at home 24 h after surgery. Self-reported parental stress levels were notably high in the group with PPOP. All 13 children had received clonidine perioperatively, but none of the children received sedative premedication. Additional details describing each child who developed PPOP are presented in Table 4.

**Table 4:** Detailed description of the 13 patients with PPOP ≥4. Sedative premedication includes clonidine, dexmedetomidine, benzodiazepines, or oxycodone.

	Surgery type, sex, and age	Preop pain and interference.	Sedative premedication	Perop analgesia	PPOP: duration, location, and interference.	PPOP according to the ICD-11 definition (9)
1	Inguinal hernia; male; 7 years old	Yes; lasting more than 2 weeks, influencing the level of activities.	No	Fentanyl bolus inf. Remifentanil; oral paracetamol preop; IV parecoxib perop; IV clonidine postop	Pain in an area other area than surgical area, enough to influence activities, occurring every week, and requiring analgesics.	No
2	Inguinal hernia; male; 1 year old	Yes; lasting more than 3 months, influencing the level of activities.	No	Inf alfentanil; ileoinguinal block; oral paracetamol preop; IV parecoxib perop; IV clonidine postop	In the area of surgery but does not influence the level of activities or require analgesics.	No
3	Inguinal hernia; female; 4 years old	No	No	Fentanyl bolus; sacral block; IV paracetamol perop; IV parecoxib perop; I v clonidine	In the area of surgery. Does not influence the level of activity. Requires analgesics.	No
4	Inguinal hernia; male; 1 year old	No	No	Remifentanil; LA by surgeon; oral paracetamol preop; IV parecoxib perop; IV clonidine postop	In the area of surgery. Does not occur every week, but when it does, it influences the level of activity and is treated with analgesics.	Yes
5	ENT; female; 15 years old	Yes; lasting more than 3 months and influencing the level of activity.	No	Fentanyl bolus; IV paracetamol; IV parecoxib; Oxy/mo bolus before wake-up; IV clonidine; +rescue opioid	In the area of surgery. Influences the level of activity. Does not occur every week. No analgesics used.	No
6	ENT; male; 1 year old	Yes; lasting more than 3 months and influencing the level of activity.	No	Alfentanil bolus; inf. Alfentanil; IV paracetamol; IV parecoxib; Oxy/mo bolus before wake-up; IV clonidine	In the area of surgery. Influences the level of activity. Occurs every week, and analgesics are used.	No
7	ENT; female; 2 years old	No	No	Fentanyl bolus; IV paracetamol; IV parecoxib; Oxy/mo bolus before wake-up; IV clonidine; +rescue opioid	Pain is not in the surgical area and does not influence the level of activity.	No
8	ENT; male; 1 year old	No	Oxy	Bolus alfentanil; inf. alfentanil; IV paracetamol; rectal ibuprofen; IV clonidine	In the area of surgery. Does not influence the level of activity. Occurs rarely and does not require analgesics.	No
9	Fracture; male; 16 years old	No	No	Inf. remifentanil; saphenous block; oral paracetamol preop; Oxy/mo bolus before wake-up; IV clonidine	In the area of surgery but does not influence the level of activity or require analgesics.	No
10	Fracture; male 9 years old	No	No	Fentanyl bolus; alfentanil bolus; inf. remifentanil; IV paracetamol; IV parecoxib; Oxy/mo bolus before wake-up; IV clonidine	In the area of surgery, with daily pain influencing the level of activities. No analgesics used.	Yes
11	Fracture; male; 16 years old	No	No	Inf. Remifentanil; IV paracetamol; IV parecoxib; Oxy/mo bolus before wake-up; IV clonidine	In the area of surgery, with weekly pain influencing the level of activities or require analgesics.	Yes
12	Hypospadias; male; 2 years old	No	Oxy	Remifentanil bolus; penile block; IV paracetamol; IV parecoxib; IV clonidine	Not in the area of surgery; does not influence the level of activities or require analgesics.	No
13	Circumcision; male; 1 year old	No	No	Penile block; IV paracetamol; IV clonidine; +rescue opioid	In the area of surgery but does not influence the level of activity or require analgesics.	No

ENT, ear nose and throat surgery (adenotonsillectomy/tonsillectomy/adenectomy); PPOP, persistent postoperative pain; LA, local anesthesia; Oxy, Oxycodone; Mo, morphine.

## Discussion

In this large prospective observational multicenter study, we found unexpectedly low levels of PPOP. Only 13 of 380 children (3.4%) reported pain ≥4 at either 3 or 6 months after surgery. The few previous studies published have suggested that PPOP in children may be lower than in adults. A prospective study of similar size by Mosetti et al. (2018) reported prevalences of 6% at 3 months and 4% at 6 months (6). In contrast, a recent prospective study including only children aged 4–7 years found much higher rates, 35% at 3 months and 27% at six months (8), approaching those reported in adults (1). Thus, the true prevalence remains uncertain, and our study contributes additional data to this evolving field.

The definition of PPOP is becoming increasingly standardized, particularly through the ICD-11 criteria and recent clarification in the BJA (9), now widely applied in adult studies (1). According to this definition, PPOP requires pain persisting for more than 3 months after surgery, but also requires that the pain begins or worsens after surgery, is located in the surgical area, is associated with pain-related distress, and interferes with daily activities (1, 9). Applying this stricter definition to our cohort resulted in an even lower prevalence: only 3 children (0.8%) experienced pain ≥ 4 lasting more than 3 months that began or increased after surgery, interfered with activity, and was localized to the surgical site. As one factor of the definition, pain-related distress, was not measured in this study our ICD-11 definition is incomplete. The actual number of children with PPOP according to ICD-11 may be even fewer than three.

A potential explanation for the very low prevalence of PPOP in our study relates to the type of surgery and the distribution of known risk factors. In adults, consistent predictors of PPOP include younger age, psychological factors (fear, depression, and pain catastrophizing), preoperative pain, and higher levels of or prolonged APOP (1). In children, however, older age seems to be a more consistent risk factor (8). Our cohort was predominantly young, with a mean age of 5.4 years, which may have contributed to the low rates of PPOP observed. The exclusion of major surgical procedures may explain the low prevalence of PPOP in this study. However, considering that the included surgeries are the most common among the pediatric population, we still consider the findings important.

Regarding other risk factors, 11% of children presented with preoperative pain, most with pain lasting more than 3 months and severe enough to limit activity. Interestingly, more children had preoperative pain than later developed PPOP, and these were largely not the same individuals: only four children with preoperative pain went on to experience PPOP. In fact, surgery appeared to improve the pain situation for many of these children. It is interesting to consider that presurgical pain has previously been found to be the only risk factor predicting PPOP at the meta-analytic level (10). In the missing value analysis, preoperative pain was found to be slightly more common in the non-responders, possibly influencing the results found here which may have rendered an underestimation of PPOP prevalence. Neither preoperative anxiety nor sleep problems appear to influence PPOP. Although 33% of children had a history of anxiety or sleep disturbance before surgery, this did not seem to lead to higher rates of PPOP in our cohort. Preoperative pain and parental stress were the only risk factors significant in the regression analysis. As parental stress was assessed at the same time as the outcome measures, a temporal relationship cannot be established, and reverse causation cannot be excluded. Therefore, causality cannot be determined. Given the very small number of children who developed PPOP, the potential for evaluation of risk factors in this study was limited. The low event rate prevents us from drawing reliable conclusions, and any associations should be interpreted with caution, as also indicated by the wide confidence intervals. For example, the risk factor APOP at 24 h has a wide confidence interval, indicating that the result is imprecise. With this caveat noted, high parental stress has previously been proposed as the most important risk factor for persistent pain in children, with previous studies finding parental pain catastrophizing to be linked to pain 3 months after surgery (8, 21).

We found it informative to closely examine the characteristics of the few children who did develop PPOP. Among the 13 affected children, several patterns emerged. Although older age is often considered a risk factor, at least for APOP, 8 of the 13 were younger than 6 years. None had PPOP ≥ 4 at both the 3- and 6-month follow-ups, even though half responded at both time points, further suggesting that the clinical relevance of their PPOP was limited. Four of the 13 children had undergone open inguinal hernia repair, of whom two had received a regional block, leaving open the question of whether inguinal hernia surgery may be associated with a higher risk of PPOP even though other studies suggest it to be less important than in adults (27). Most children responding to the follow-up did not report preoperative pain, but the four who did all described pain severe enough to interfere with daily activities.

APOP did not appear to be a major contributor in this group. Only three children experienced breakthrough pain in the PACU, and all but one legal guardian felt that their child received adequate analgesia during and after surgery. Ten children had a pain score of 0 recorded in the PACU, and three required rescue opioids. Only one child reported APOP ≥ 4 at home 24 h after surgery.

This is one of the few prospective observational studies looking at PPOP in children after routine pediatric surgery. Its multicenter design, large sample size, and inclusion of children across the full pediatric age range (1–17 years) make the findings representative of a common Swedish pediatric surgical cohort. We looked at all aspects required to thoroughly evaluate PPOP according to current standards, and our results indicate that very few children develop persistent pain after surgery.

There are several limitations to this study. The most important concerns inclusion. Although we aimed to enroll 1,000 children, practical challenges, in mainly obtaining oral and written consent from both legal guardians, as required by the Swedish Ethics Review Authority, resulted in the inclusion of only 601 children within the study period. Of these, 380 responded to at least one follow-up questionnaire at 3 or 6 months, yielding a response rate of 63%.

Another limitation relates to the use of questionnaire-based follow-up, in which pain assessments for all children under 15 years were completed by legal guardians. While in-person clinical assessments would have been ideal, they were not feasible for a cohort of this size.

Although the ICD-11 provides a clearer framework for defining PPOP, it does not specify a minimum pain score that should be considered clinically meaningful. We used a threshold of ≥4, consistent with the most recent pediatric studies, to allow for comparison. However, we acknowledge that pain scores below 4 may still be significant for individual children. In our cohort, eight children registered PPOP at either 3 or 6 months at a level lower than four but still influencing their level of activity; five of these children had pain in the area of surgery, but only one child had pain daily. We anticipate future work, as in adults, on suitable core outcome sets for postoperative pain and minimal clinically important changes (28) for children as well.

There is also no universal consensus on which pain scale is most appropriate for each age group. The NRS could have been used for the evaluation of pain from 8 years of age, specifically concerning PPOP and chronic pain (29). As there are no strong recommendations, this limit was set due to technical issues, with the FPS-R being used as long as the evaluations were recorded by the caregiver, and because school children are used to using faces in school evaluations.

A further limitation is the unexpectedly low prevalence of PPOP, 3% rather than the anticipated 20%, which restricts the reliability of risk factor analysis. For example, although preoperative pain and parental stress appeared significant in the regression model, the wide confidence intervals indicate that these findings should be interpreted cautiously. As such, this study should be viewed primarily as descriptive and hypothesis-generating.

Despite the small number of children with PPOP, we examined this subgroup in detail to explore potential patterns. However, we did not identify a clear explanation for why some children develop PPOP while most do not, except possibly the influence of parental stress levels.

## Conclusion

In this cohort of children undergoing common pediatric surgeries, only six out of 380 children developed pain that interfered with their daily activities, five required any form of analgesics at 3–6 months, and only one child experienced daily pain. The number of children with PPOP was too small for reliable statistical analysis of risk factors. When examining these cases descriptively, parental stress may have been one possible factor contributing to the development of PPOP in these children. In the future, even larger prospective studies are essential to capture enough cases of children developing PPOP to allow robust statistical analysis of risk factors which will be crucial for understanding the mechanisms behind PPOP and for developing targeted strategies to prevent it.

## Data Availability

The raw data supporting the conclusions of this article will be made available by the authors, without undue reservation.

## References

[1] Rosenberger DC Pogatzki-Zahn EM. Chronic post-surgical pain - update on incidence, risk factors and preventive treatment options. BJA Educ. (2022) 22(5):190–6. 10.1016/j.bjae.2021.11.008 35496645 PMC9039436

[2] Rabbitts JA Fisher E Rosenbloom BN Palermo TM. Prevalence and predictors of chronic postsurgical pain in children: a systematic review and meta-analysis. J Pain. (2017) 18(6):605–14. 10.1016/j.jpain.2017.03.007 28363861 PMC5457338

[3] Sieberg CB Simons LE Edelstein MR DeAngelis MR Pielech M Sethna N. Pain prevalence and trajectories following pediatric spinal fusion surgery. J Pain. (2013) 14(12):1694–702. 10.1016/j.jpain.2013.09.005 24290449 PMC3873090

[4] Page MG Stinson J Campbell F Isaac L Katz J. Identification of pain-related psychological risk factors for the development and maintenance of pediatric chronic postsurgical pain. J Pain Res. (2013) 6:167–80. 10.2147/JPR.S40846 23503375 PMC3594915

[5] Batoz H Semjen F Bordes-Demolis M Benard A Nouette-Gaulain K. Chronic postsurgical pain in children: prevalence and risk factors. A prospective observational study. Br J Anaesth. (2016) 117(4):489–96. 10.1093/bja/aew260 28077537

[6] Mossetti V Boretsky K Astuto M Locatelli BG Zurakowski D Lio R. Persistent pain following common outpatient surgeries in children: a multicenter study in Italy. Paediatr Anaesth. (2018) 28(3):231–6. 10.1111/pan.13321 29352738

[7] Fortier MA Chou J Maurer EL Kain ZN. Acute to chronic postoperative pain in children: preliminary findings. J Pediatr Surg. (2011) 46(9):1700–5. 10.1016/j.jpedsurg.2011.03.074 21929977

[8] Ceniza-Bordallo G Fraile AG López-de-Uralde-Villanueva I Rabbitts JA Li R Palermo TM. Chronic postsurgical pain in young children: prevalence, pain trajectories and physical and psychological prognostic factors. J Pain. (2025) 34:105476. 10.1016/j.jpain.2025.105476 40562263 PMC12282272

[9] Stamer UM Lavand'homme P Hofer DM Barke A Korwisi B. Definition of chronic postsurgical pain in the ICD-11: a narrative review of implications for anaesthesiology and pain medicine. Br J Anaesth. (2026) 136(4):1319–29. 10.1016/j.bja.2025.02.005 40089399

[10] Rosenbloom BN Frederiksen SD Wang V Birnie KA Park CS Gordon G. Prevalence of and recommendation for measuring chronic postsurgical pain in children: an updated systematic review and meta-analysis. Reg Anesth Pain Med. (2025) 50(2):132–43. 10.1136/rapm-2024-105697 39909546 PMC11804871

[11] King S Chambers CT Huguet A MacNevin RC McGrath PJ Parker L. The epidemiology of chronic pain in children and adolescents revisited: a systematic review. Pain. (2011) 152(12):2729–38. 10.1016/j.pain.2011.07.016 22078064

[12] Walther-Larsen S Pedersen MT Friis SM Aagaard GB Rømsing J Jeppesen EM. Pain prevalence in hospitalized children: a prospective cross-sectional survey in four Danish university hospitals. Acta Anaesthesiol Scand. (2017) 61(3):328–37. 10.1111/aas.12846 28032329

[13] Groenewald CB Rabbitts JA Schroeder DR Harrison TE. Prevalence of moderate-severe pain in hospitalized children. Paediatr Anaesth. (2012) 22(7):661–8. 10.1111/j.1460-9592.2012.03807.x 22332912

[14] Vejzovic V Bozic J Panova G Babajic M Bramhagen AC. Children still experience pain during hospital stay: a cross-sectional study from four countries in Europe. BMC Pediatr. (2020) 20(1):39. 10.1186/s12887-020-1937-1 31996162 PMC6988252

[15] Alm F Stalfors J Nerfeldt P Ericsson E. Patient reported pain-related outcome measures after tonsil surgery: an analysis of 32,225 children from the national tonsil surgery register in Sweden 2009–2016. Eur Arch Otorhinolaryngol. (2017) 274(10):3711–22. 10.1007/s00405-017-4679-4 28815308 PMC5591799

[16] Ferland CE Vega E Ingelmo PM. Acute pain management in children: challenges and recent improvements. Curr Opin Anaesthesiol. (2018) 31(3):327–32. 10.1097/ACO.0000000000000579 29432292

[17] Smeland AH Rustøen T Næss T Nybro L Lundeberg S Reinertsen H. Children's views on postsurgical pain in recovery units in Norway: a qualitative study. J Clin Nurs. (2019) 28(11-12):2157–70. 10.1111/jocn.14788 30667569

[18] Page MG Stinson J Campbell F Isaac L Katz J. Pain-related psychological correlates of pediatric acute post-surgical pain. J Pain Res. (2012) 5:547–58. 10.2147/JPR.S36614 23204864 PMC3508661

[19] Chow CHT Schmidt LA Buckley DN. The role of anxiety and related states in pediatric postsurgical pain. Can J Pain. (2020) 4(4):26–36. 10.1080/24740527.2020.1847600 33987517 PMC7942768

[20] Beveridge JK Noel M Soltani S Neville A Orr SL Madigan S. The association between parent mental health and pediatric chronic pain: a systematic review and meta-analysis. Pain. (2024) 165(5):997–1012. 10.1097/j.pain.0000000000003125 38112571

[21] Page MG Campbell F Isaac L Stinson J Katz J. Parental risk factors for the development of pediatric acute and chronic postsurgical pain: a longitudinal study. J. Pain Res. (2013) 6:727–41. 10.2147/JPR.S51055 24109194 PMC3792832

[22] Broman J Nielsen N Persson AKM. A prospective observational study on persistent postoperative pediatric pain, 4P: the study protocol. PLoS One. (2025) 20(1):e0316533. 10.1371/journal.pone.0316533 39808613 PMC11731717

[23] Manworren RC Hynan LS. Clinical validation of FLACC: preverbal patient pain scale. Pediatr Nurs. (2003) 29(2):140–6. 12723828

[24] Hicks CL Von Baeyer CL Spafford PA Van Korlaar I Goodenough B. The faces pain scale-revised: toward a common metric in pediatric pain measurement. Pain. (2001) 93(2):173–83. 10.1016/S0304-3959(01)00314-1 11427329

[25] Von Baeyer CL Spagrud LJ McCormick JC Choo E Neville K Connelly MA. Three new datasets supporting use of the numerical rating scale (NRS-11) for children's self-reports of pain intensity. Pain. (2009) 143(3):223–7. 10.1016/j.pain.2009.03.002 19359097

[26] Vittinghoff M Lönnqvist P-A Mossetti V Heschl S Simic D Colovic V. Postoperative pain management in children: guidance from the pain committee of the European society for paediatric anaesthesiology (ESPA pain management ladder initiative). Paediatr Anaesth. (2018) 28(6):493–506. 10.1111/pan.13373 29635764

[27] Kristensen AD Ahlburg P Lauridsen MC Jensen TS Nikolajsen L. Chronic pain after inguinal hernia repair in children. Br J Anaesth. (2012) 109(4):603–8. 10.1093/bja/aes250 22831892

[28] Vollert J Egert VM Segelcke D Fletcher D Kalso E Kemp H. Minimal clinically important changes of patient-reported outcome measures for acute postsurgical pain. Anesthesiology. (2026) 144(1):143–53. 10.1097/aln.0000000000005792 41065659 PMC12677341

[29] Birnie KA Hundert AS Lalloo C Nguyen C Stinson JN Recommendations for selection of self-report pain intensity measures in children and adolescents: a systematic review and quality assessment of measurement properties. Pain. 2019;160(1):5–18. 10.1097/j.pain.0000000000001377 30180088

